# *log*(TG)/HDL-C is related to both residual cardiometabolic risk and β-cell function loss in type 2 diabetes males

**DOI:** 10.1186/1475-2840-9-88

**Published:** 2010-12-14

**Authors:** Michel P Hermans, Sylvie A Ahn, Michel F Rousseau

**Affiliations:** 1 Endocrinology and Nutrition Unit, Cliniques Universitaires St-Luc, Avenue Hippocrate 10, 1200 Brussels, Belgium; 2 Division of Cardiology, Cliniques universitaires St-Luc, Avenue Hippocrate 10, 1200 Brussels, Belgium

## Abstract

**Background:**

T2DM is associated with atherogenic dyslipidemia (AD), defined as decreased HDL-C plus raised triglycerides (TG). AD confers increased risk for CAD, even when LDL-C is at target. AD is rarely assessed due to lack of screening methods consensus.

**Aim:**

To establish the prevalence and severity of AD from *log*(TG)/HDL-C in T2DM males, and to determine how it relates to cardiometabolic phenotype, glucose homeostasis, micro- and macrovascular complications, and 10-year *UKPDS *CV risk.

**Methods:**

585 T2DM males divided according to quintiles (Q) of *log*(TG)/HDL-C. AD prevalence defined as HDL-C <40 mg.dL^-1 ^plus TG ≥150 mg.dL^-1^. β-cell function assessed with HOMA.

**Results:**

Mean HDL-C and TG were 44 (13) and 204 (155) mg.dL^-1^. AD prevalence was 35%. AD correlated with lower β-cell function, with accelerated loss of insulin secretion, and with poorer HbA_1c _levels. AD was related to a high prevalence of CAD, and also to 10-year absolute CAD risk.

**Conclusions:**

*log*(TG)/HDL-C is a simple means to estimate AD and the residual CV risk it confers in T2DM. AD closely associates with major cardiometabolic and glucose homeostasis determinants and poorer metabolic control. The ratio also relates to macroangiopathy prevalence and ranks future CAD risk, and is well-suited to capture non-LDL-related macrovascular residual risk and major glycemic determinants.

## Introduction

Current guidelines recommend aggressive treatment of low-density lipoprotein cholesterol (LDL-C) in patients with type 2 diabetes mellitus (T2DM) with statins as preferred agent [1]. Besides hypercholesterolemia, T2DM is associated with a specific non-LDL dyslipidemia, known as atherogenic dyslipidemia (AD). The hallmark of AD consists of decreased high-density lipoprotein cholesterol (HDL-C) together with raised triglycerides (TG). AD is associated with insulin resistance (IR), and confers a marked increase in residual vascular risk, even when LDL-C is low [2-9].

Screening for AD may provide clinically relevant information for assessing residual vascular risk associated with the common determinants of low HDL-C and high TG. However, this is rarely performed in practice, due to lack of agreement criteria or consensual cut-offs to routinely establish and grade AD based on measurements of HDL-C and fasting TG levels. Low HDL-C and high TG are part of the metabolic syndrome (MetS) definition, either as single AD components or in combination. Cordero *et al. *proposed gender-specific ratio's thresholds to correlate with MetS in non-diabetics [10]. Yet, despite their usefulness for diagnosing MetS and AD, low HDL-C and high TG are often dealt as separate, second-rank modifiable CV risk factors [5,6,11,12].

A more comprehensive approach defines AD by the *combined *occurrence of high TG levels and low HDL-C. Yet, such seemingly easy estimation is hampered by (*i*) lack of consensual cut-off values across gender, ethnicities and underlying conditions (*ii*) requirement for baseline lipid values prior to lipid-lowering drugs (LLD), (*iii*) a *sine qua non *association does not take into account imbalances between the severity of the two non-LDL lipid abnormalities, underestimating the magnitude of AD in groups with spontaneously low (Afro-Americans, sub-Saharan Africans) or with elevated TG levels prior to LLD or insulin [12-16].

Computing a *ratio *from fasting TG (*numerator*) and fasting HDL-C (*denominator*) appears as such intuitively more attractive than the combined occurrence to grade AD severity. Prior *log *transformation of TG levels allows for computing a broad range of TG values, as observed in everyday practice, from normal levels in subjects without AD to markedly hypertriglyceridemic states, such as MetS and many T2DM patients. Assessing AD with a *log*(TG)/HDL-C ratio also incorporates occurrence of mutually reinforcing or diverging confounders affecting both fraction's components, such as insulin-sensitizers, certain LLDs, or ethanol intake (which often increase both terms), or diet, exercise or menopausal status, which affect numerator and denominator in opposite directions [12,17-20].

The aim of the present study was to establish the prevalence, distribution and severity of AD from *log*(TG)/HDL-C in a large cohort of T2DM males. We also determined whether the ratio provides predictive information on cardiometabolic phenotype and glucose homeostasis determinants. Finally, we analyzed the association between AD and prevalence of micro- and macrovascular complications, as well as its impact on 10-year CV risk from the T2DM-specific *United Kingdom Prospective Diabetes Study *(UKPDS) calculator [21-25].

## Methods

The study design was cross-sectional and included 585 consecutive adult male outpatients with T2DM defined according to the *Expert Committee on the Diagnosis and Classification of Diabetes Mellitus *[26] and followed in a single academic centre in Brussels (Belgium) between January 2008 and June 2010. Exclusion criteria were other forms of diabetes, severely impaired hepatic function, cancer, untreated thyroid conditions associated with hypo- or hyperthyroidism, malignancies and malabsorptive gastro-intestinal disorders. The following variables were recorded: age, ethnicity, socio-educational level, known diabetes duration, family history (cardiovascular diseases, diabetes mellitus), self-reported leisure-time (LT) weekly exertional exercise duration and daily duration spent watching screen(s) such as television, personal computer or visual numeric media, as surrogate for leisure-time sedentarity.

The presence of a metabolic syndrome (MetS score ≥3/5) was defined according to *AHA/NHLBI *criteria [11,16]. The prevalence of AD was defined as the combination of MetS criteria for low HDL-C at baseline (< 40 mg.dL^-1^) and high fasting TG at baseline (≥150 mg.dL^-1^). The AD ratio *log*(TG)/HDL-C computed *log*(fasting TG) to fasting HDL-C. In patients treated with LLD(s), the TG and HDL-C values were the last available ones prior to LLD implementation. Normal values for *log*(TG)/HDL-C from a control cohort of 80 lean subjects without diabetes or family history for diabetes and untreated with LLD were: 0.036 (*mean*); 0.034 (*median*); 0.012 (*SD*); 0.014 (*minimum*); 0.067 (*maximum*); 0.029 (*percentile *25) and 0.042 (*percentile *75).

As regards bioanthropometry, patients were measured for body weight, height, body mass index (BMI), relative and total body fat (BodyFat Analyzer, Omron BF 500), waist circumference and conicity index (waist circumference (m)/0.109 √[weight (kg)/height (m)]) [27-29]. Ultrasonographic evidence for fatty liver was considered in the presence of hyperreflectivity and in the absence of other etiological factors known to be associated with liver steatosis, including excess ethanol intake.

Computer-based Homeostasis Model Assessment (HOMA) of insulin sensitivity and beta-cell function was previously detailed http://www.dtu.ox.ac.uk. Values of HOMA B (%) were plotted as a function of HOMA S (%), defining a hyperbolic product area (β × S) [unit: %^2^; normal value: 100%, corresponding to 10^4 ^%^2^], which represents the true, underlying β-cell function. (B × S) loss over a subject's lifetime span was obtained by dividing 100-(B × S) by subjects' age at the time of HOMA-modeling, providing an estimate of annual (B × S) loss rate (%.year^-1^) [15,30-34].

Medications history inquired upon current use of oral antidiabetic drugs, insulin, blood pressure (BP)-lowering drugs, aspirin, and any LLD(s)). Hypertension history was defined as systolic BP ≥140 mmHg and/or diastolic BP ≥90 mmHg or current treatment with BP-lowering drug(s) prescribed for treating high BP.

As regards macroangiopathy, coronary artery disease (CAD) was retrospectively inferred from medical history (myocardial infarction, angioplasty, stenting, revascularization surgery and/or significant coronary stenosis confirmed by angiography) and systematic review of all procedures, screening (exercise testing, echocardiography) or subclinical disease imaging data in the patient's records. Stroke was defined according to UK Prospective Diabetes Study (UKPDS) criteria, i.e. any neurological deficit lasting ≥1 month, no distinction being made between ischaemic, embolic and haemorrhagic strokes [21]. In patients with multiple CV events, only the first one was considered for prevalence. Peripheral artery disease (PAD) was defined by a well-documented medical history of lower-limb(s) claudication and/or clinical or imaging evidence for ischemic diabetic foot, angioplasty, stenting, revascularization surgery and/or significant lower-limb artery stenosis at Doppler ultrasonography and/or angiography. A diabetic retinopathy (DRP) was diagnosed following dilated fundus examination, with fluoangiography performed when deemed necessary by an in-house ophthalmologist. The presence of a peripheral neuropathy was based on clinical examination (knee and ankle reflexes, Semmes-Weinstein 5.07 monofilament test) and/or 4-limbs electromyography.

The following biologic variables were assessed: glycated haemoglobin (HbA_1c_), total cholesterol (C), HDL-C, TG, LDL-C (from Friedewald's formula), non-HDL-C (by subtracting HDL-C from total C), apolipoproteins (apo) A-I and B_100 _. As for non-lipid cardiometabolic markers, levels of high-sensitivity C-reactive protein (_hs_CRP), uric acid, cystatin C, fibrinogen, leucocytes count, total and free testosterone, sex-hormone-binding globulin (SHBG), ferritin, liver enzymes (*aspartate aminotransferase *[AST], *alanine aminotransferase *[ALT], *γ-glutamyl transferase *[γGT], homocysteine, folic acid and vitamin B_12 _were determined. Normo, micro- and macro-albuminuria were defined as urinary albumin excretion <30 (normo-), 30-299 (microalbuminuria) and ≥300 μg.mg creatinine^-1 ^(macro-albuminuria) from first-morning urine sample. Glomerular filtration rate was estimated (eGFR) using the *Modified Diet in Renal Disease *(MDRD) formula [35].

The *UKPDS Risk Engine *provided 10-year absolute CV risk estimates for T2DM individuals in primary CV prevention related to coronary artery disease (CAD), lethal CAD, stroke and lethal stroke, and was based on the 10 following variables: *known T2DM duration, age, gender, ethnicity, smoking status, chronic atrial fibrillation, HbA_1c _level, systolic BP, total C *and *HDL-C *[21-25]. Each patient gave informed consent, and the protocol was approved by the local Institutional Review Board.

### Statistical methods

Results are presented as means (± 1 standard deviation (SD)) or proportions. The significance of differences between means was assessed by one-way analysis of variance for linear trend between means, and by a Chi-squared test trend for differences in proportions. Results were considered significant or non-significant (NS) for p < or > 0.05, respectively.

## Results

Patient's characteristics are described in Table 1. There were 585 male patients in the whole cohort, 89% of whom of Caucasian ancestry. Diabetes duration was 14 (9) years. The prevalence of AD, defined as the combination of baseline HDL-C <40 mg.dL^-1 ^and TG ≥150 mg.dL^-1 ^was 35%. When patients were divided according to AD quintiles of *log*(TG)/HDL-C, patients in the 1^st ^AD quintile (Q I) had a mean *log*(TG)/HDL-C value similar to that of control, non-diabetic subjects (see *Methods*). T2DM patients from the 2^nd ^AD quintile (Q II) had a mean *log*(TG)/HDL-C value corresponding to the 75^th ^percentile of controls.

**Table 1:** Patients' characteristics

		All patients	Q I	Q II	Q III	Q IV	Q V	*P*
***n***		**585**	**117**	**117**	**117**	**117**	**117**	
								
***log*(TG). HDL-C^-1^**		**0.053**	**0.032**	**0.042**	**0.050**	**0.060**	**0.082**	*~*
**age**	years	**65 (12)**	**67 (12)**	**67 (12)**	**63 (12)**	**65 (12)**	**63 (10)**	*0.0083*
smoking *	%	**36/64**	**41/59**	**44/56**	**40/60**	**31/69**	**24/76**	*0.0010*
**ethanol**	U.week^-1^	**16 (23)**	**21 (25)**	**19 (21)**	**16 (24)**	**14 (21)**	**11 (25)**	*0.0093*
LT physical activity **	%	**60/40**	**55/45**	**53/47**	**58/42**	**67/33**	**68/32**	*0.0072*
LT screen watching ***	hr.day^-1^	**3.1 (1.8)**	**2.7 (1.8)**	**2.8 (1.6)**	**2.8 (1.6)**	**3.0 (1.5)**	**4.0 (2.2)**	*<0.0001*
**BMI**	kg.m^-2^	**28.8 (5.1)**	**26.5 (4.9)**	**27.9 (4.5)**	**29.2 (5.0)**	**30.1 (5.0)**	**30.4 (5.3)**	*<0.0001*
**waist circumference**	cm	**105 (13)**	**98 (13)**	**103 (11)**	**105 (12)**	**109 (12)**	**109 (13)**	*<0.0001*
**conicity index**	m^2^.kg^-1^	**1.35 (0.08)**	**1.33 (0.08)**	**1.35 (0.07)**	**1.36 (0.08)**	**1.37 (0.07)**	**1.37 (0.08)**	*0.0002*
**fat mass**	%	**27.4 (6.2)**	**25.0 (6.7)**	**26.9 (6.0)**	**27.5 (6.5)**	**28.6 (5.5)**	**28.8 (5.6)**	*<0.0001*
**visceral fat**	0 - 30	**14 (5)**	**12 (5)**	**13 (4)**	**14 (5)**	**15 (4)**	**15 (5)**	*<0.0001*
**liver steatosis**	%	**68**	**50**	**61**	**69**	**72**	**83**	*<0.0001*
**insulinaemia**	pmol.l^-1^	**113 (77)**	**80 (47)**	**103 (67)**	**122 (98)**	**125 (83)**	**132 (71)**	*<0.0001*
**HOMA S**	%	**53 (34)**	**69 (39)**	**61 (40)**	**50 (30)**	**49 (30)**	**39 (23)**	*<0.0001*
**HOMA product (B × S)**	%	**28.5 (18.2)**	**35.7 (21.6)**	**31.1 (16.2)**	**26.7 (15.8)**	**26.7 (16.2)**	**22.5 (16.5)**	*<0.0001*
**(B × S) loss rate**	%.yr^-1^	**1.29 (0.45)**	**1.15 (0.50)**	**1.18 (0.33)**	**1.38 (0.52)**	**1.28 (0.40)**	**1.44 (0.39)**	*<0.0001*
**metabolic syndrome**	%	**76**	**36**	**67**	**83**	**95**	**97**	*<0.0001*
**metabolic syndrome score**	1/5 to 5/5	**3.4**	**2.3**	**2.9**	**3.5**	**4.0**	**4.4**	*<0.0001*
**statin/fibrate**	%	**54/21**	**49/9**	**51/11**	**59/21**	**57/16**	**56/46**	*NS/<0.0001*
**macroangiopathy**	%	**40**	**29**	**38**	**44**	**40**	**48**	*0.006*
**CAD**	%	**29**	**15**	**32**	**32**	**31**	**33**	*0.0097*
**TIA/stroke**	%	**9**	**5**	**5**	**10**	**10**	**13**	*0.0129*
**PAD**	%	**12**	**15**	**13**	**11**	**8**	**14**	*NS*

Results are expressed as means (1 SD) or proportions (%). *: never vs. former/current; **: no leisure-time (LT) physical activity (PA) vs. any level of LT-PA. CAD: coronary artery disease; BMI: body mass index; HOMA B & S: beta-cell function and insulin sensitivity from homeostatic model assessment; PAD: peripheral artery disease; Q: quintile; TIA: transient ischaemic attack. NS: not significant.

In the whole T2DM cohort, mean age (1 SD) was 65 (12) year, with a significant downward trend across AD quintiles. Both smoking and ethanol intake showed significant decreasing trends across quintiles. The proportion of patients reporting LT physical activity and screen-watching daily duration were significantly different across quintiles, suggestive of both a marked increase in sedentarity and a marked decrease in exercise practice. There were highly-significant trends across AD quintiles for progressively higher values of BMI, waist circumference, conicity index, waist-to-height ratio, fat mass and visceral fat. Liver steatosis was present in 68% of the whole cohort, with significant rising trends across quintiles, from 50% (Q I) to 83% (Q V) (p < 0.0001). There was a stepwise increase across quintiles of *log*(TG)/HDL-C in fasting insulinemia, from 80 to 132 pmol.l^-1 ^(p < 0.0001). Insulin sensitivity and the hyperbolic product (B × S) were lower than the normal value (100%) in the whole cohort, with a mean value of 53% (HOMA S) and 28.5% (B × S), respectively. There were stepwise decreases across quintiles for both parameters, from 69% (HOMA S) and 35.7% (B × S) (Q I) to 39% (HOMA S) and 22.5% (B × S) (Q V) (both p < 0.0001). The B × S loss rate also showed a highly-significant trend toward worsening loss across AD quintiles, from 1.15%.yr^-1 ^(Q I) to 1.44%.yr^-1 ^(Q V) (p < 0.0001; Table 1).

Statin and fibrate were given to 54 and 21% of the whole cohort, with a significant trend for increased fibrate use across quintiles, from 9% (Q I) to 46% (Q V) (p < 0.0001; Table 1). None of the patients were treated with niacin. In the whole cohort, macroangiopathy was present in 40%, as CAD (29%), TIA/stroke (9%) and/or PAD (12%) (Table 1). There were significant trends for higher prevalence of overall macroangiopathy, CAD and TIA/stroke across AD quintiles, from 29% (overall), 15% (CAD) and 5% (TIA/stroke) in Q I to 48% (overall), 33% (CAD) and 13% (TIA/stroke) in Q V (p 0.060, 0.0097 and 0.0129, respectively). No trend was observed for PAD across AD quintiles. After adjustment for inter-quintile differences in age, overall macroangiopathy, CAD and TIA/stroke prevalence increased even more significantly across quintiles, from 28%, 15% and 6% (Q I) to 52%, 36% and 14% (Q V), respectively (p 0.0010 (overall), 0.0024 (CAD), and 0.0126 (TIA/stroke)).

As regards glucose-lowering therapies, 59, 47, 7, 3, and 42% were receiving metformin, a β-cell stimulant, a glitazone, a DPP4-inhibitor and/or insulin, without relevant trends between quintiles. Mean HbA_1c _in the whole cohort was 7.53 (1.53)%, with 43% of patients at HbA_1c _target (< 7.0%). There was a non-significant rise in HbA_1c _across AD quintiles, from 7.32% (Q I) to 7.85% (Q V), and a significant decrease across AD quintiles in proportion of patients reaching target HbA_1c_, from 49% (Q I) to 35% (Q V; p 0.0155; Table 2).

**Table 2:** Laboratory values

		All patients	Q I	Q II	Q III	Q IV	Q V	*P*
***n***		**585**	**117**	**117**	**117**	**117**	**117**	
								
**HbA_1c_**	%	**7.53 (1.53)**	**7.32 (1.36)**	**7.41 (1.52)**	**7.48 (1.46)**	**7.60 (1.55)**	**7.85 (1.72)**	*NS*
**HbA_1c _< 7.0%**	%	**43**	**49**	**47**	**45**	**40**	**35**	*0.0155*
**cholesterol (C)**	mg.dl^-1^	**175 (41)**	**179 (35)**	**180 (37)**	**168 (40)**	**174 (41)**	**174 (48)**	*NS*
**LDL-C**	mg.dl^-1^	**98 (35)**	**100 (32)**	**107 (34)**	**96 (34)**	**97 (34)**	**90 (38)**	*0.0047*
**non-HDL-C**	mg.dl^-1^	**131 (40)**	**118 (33)**	**132 (36)**	**127 (38)**	**136 (40)**	**143 (47)**	*<0.0001*
**non-HDL-C - LDL-C**	mg.dl^-1^	**31 (18)**	**18 (8)**	**25 (11)**	**30 (15)**	**38 (18)**	**47 (21)**	*<0.0001*
**HDL-C**	mg.dl^-1^	**44 (13)**	**61 (11)**	**48 (8)**	**42 (7)**	**38 (6)**	**32 (8)**	*~*
**apoA1**	mg.dl^-1^	**143 (27)**	**167 (23)**	**151 (24)**	**139 (20)**	**137 (18)**	**122 (26)**	*<0.0001*
**apoB_100_**	mg.dl^-1^	**90 (26)**	**80 (23)**	**92 (26)**	**86 (24)**	**94 (25)**	**95 (30)**	*<0.0001*
**pre-LLD TG**	mg.dl^-1^	**204 (155)**	**102 (46)**	**149 (61)**	**173 (82)**	**217 (72)**	**359 (254)**	*~*
**current TG**	mg.dl^-1^	**171 (119)**	**92 (40)**	**129 (66)**	**153 (77)**	**200 (102)**	**282 (165)**	*~*
**LDL-C. apoB_100_^-1^**		**1.07 (0.28)**	**1.21 (0.24)**	**1.13 (0.26)**	**1.07 (0.26)**	**0.99 (0.28)**	**0.92 (0.27)**	*<0.0001*
**_hs_CRP**	mg.dl^-1^	**0.36 (0.61)**	**0.36 (0.73)**	**0.20 (0.21)**	**0.42 (0.72)**	**0.35 (0.44)**	**0.48 (0.76)**	*0.0089*
**uric acid**	mg.dl^-1^	**5.7 (1.6)**	**5.5 (1.4)**	**5.7 (1.5)**	**5.6 (1.7)**	**6.3 (1.6)**	**5.7 (1.6)**	*0.001*
**cystatin C**	mg.l^-1^	**0.87 (0.34)**	**0.76 (0.21)**	**0.85 (0.22)**	**0.85 (0.27)**	**0.90 (0.38)**	**0.98 (0.48)**	*<0.0001*
**white blood cells**	10^3^.mm^-3^	**7.20 (1.94)**	**6.63 (2.04)**	**6.70 (1.90)**	**7.49 (1.90)**	**7.57 (2.01)**	**7.56 (1.58)**	*<0.0001*
**ALT**	IU.l^-1^	**32 (21)**	**27 (11)**	**33 (26)**	**30 (16)**	**38 (28)**	**32 (14)**	*0.0009*
**SHBG**	nmol.l^-1^	**37 (24)**	**46 (25)**	**37 (21)**	**31 (15)**	**36 (25)**	**34 (30)**	*<0.0001*
**eGFR**	ml.min^-1^.1.73 m^2^	**80 (27)**	**85 (25)**	**80 (23)**	**82 (29)**	**81 (24)**	**73 (30)**	*0.0105*
**albuminuria**	μg.mg creatinine^-1^	**100 (278)**	**50 (103)**	**30 (55)**	**108 (273)**	**106 (304)**	**201 (433)**	*<0.0001*
**normal/(μ)albuminuria**	%	**65/35**	**76/24**	**73/27**	**64/36**	**63/37**	**51/49**	*<0.0001*

Results are expressed as means (1 SD) or proportions (%). ALT: alanine aminotransferase; apo: apolipoprotein; eGFR: estimated glomerular filtration rate; HDL: high-density lipoprotein; _hs_CRP: high-sensitivity C-reactive protein; LDL: low-density lipoprotein; LLD: lipid-lowering drug(s); μ: micro-; Q: quintile; SHBG: sex hormone-binding globulin; TG: triglycerides. NS: not significant.

**Figure 1:**
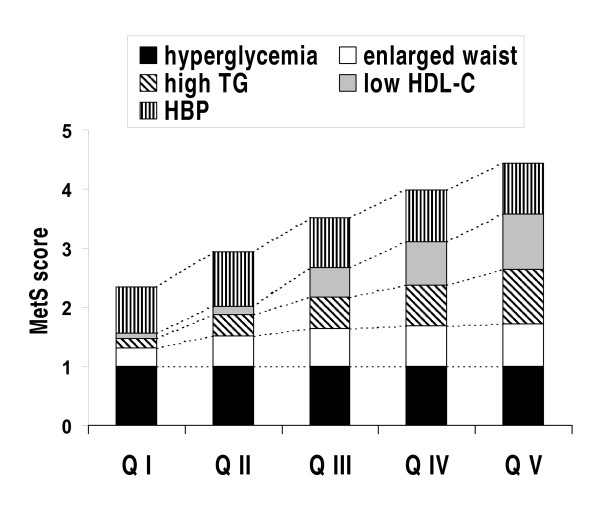
**Contribution of individual components of the metabolic syndrome (MetS) phenotype to the MetS cumulative score in 585 male patients with T2DM, divided into quintiles (*n *= 117; QI to QV) based on *log*(TG)/HDL-C ranking distribution**. HBP: high blood pressure; HDL-C: high-density lipoprotein cholesterol; TG: triglycerides.

The contribution of discrete components to the MetS score for each AD quintile is depicted on Figure 1. A MetS phenotype (score ≥3/5) was present in 76% in the whole cohort. There was a highly-significant stepwise rise in both MetS prevalence and score across AD quintiles, from 36% and 2.3 (Q I) to 97% and 4.4 (Q V) (both p < 0.0001; Table 1 and Figure 1). There were no significant trends for higher prevalence of hypertension or enlarged waist prevalence across quintiles. In the whole cohort, hypertension prevalence was 86% and mean systolo-diastolic blood pressure values were 139 (19) - 80 (11) mmHg, without trends across quintiles.

In the whole cohort, mean LDL-C was 98 (35) mg.dL^-1^, HDL-C was 44 (13) mg.dL^-1^, and current TG levels were 171 (119) mg.dL^-1^. Baseline, pre-LLD TG values were 204 (155) mg.dL^-1^. Non-HDL-C was on average 131 (40) mg.dL^-1^, and the delta between non-HDL-C and LDL-C was 31 (18) mg.dL^-1^, suggesting elevated number of both apoB_100_-carrying particles and TG-rich lipoproteins enrichment. There were significant trends for decreasing values across AD quintiles for LDL-C, apoA-I, and LDL-C/apoB_100 _ratio as surrogate for LDL size (p 0.0047, < 0.0001, and < 0.0001, respectively). There were significant trends as well for increasing values across AD quintiles for non-HDL-C, non-HDL-C *minus *LDL-C, and apoB_100 _(all p < 0.0001; Table 2).

As regards non-lipids markers, there were significant trends for increasing levels across quintiles for _hs_CRP, uric acid, cystatin C, leucocytes and ALT. Significant decreases across quintiles were observed for SHBG and vitamin B_12 _levels (p < 0.0001) (Table 2) as well as for total testosterone (11.5 (4.4) [Q I], 11.2 (5.9) [Q II], 10.6 (4.1) [Q III], 9.7 (3.9) [Q IV] and 10.4 (4.9) nmol.L^-1 ^[Q V] (p 0.0331)). Mean values for the non-lipid parameters in the whole cohort were: fibrinogen 316 (76) mg.dL^-1^, ferritin 206 (189) ng.mL^-1^, free testosterone 0.19 (0.12) nmol.L^-1^, homocysteine 14.1 (6.9) μmol.L^-1^, folic acid 7.7 (5.2) ng.mL^-1^, AST 26 (11) IU.L^-1^, and γGT 43 (48) IU.L^-1^, with no trends across quintiles.

Diabetic retinopathy and peripheral polyneuropathy were diagnosed in 22 and 30% of the whole cohort, with no trend for higher prevalence across AD quintiles. Such lack of difference across quintiles remained after adjustment for inter-quintile differences in mean age. In the whole cohort, eGFR was 80 (27) ml.min^-1^.1.73 m^2^, with a significant decrease across AD quintiles, from 85 (Q I) to 73 ml.min^-1^.1.73 m^2 ^(Q V; p 0.0105). There were significant increases across quintiles in mean albuminuria values and in albuminuria prevalence (both p < 0.0001; Table 2).

**Figure 2:**
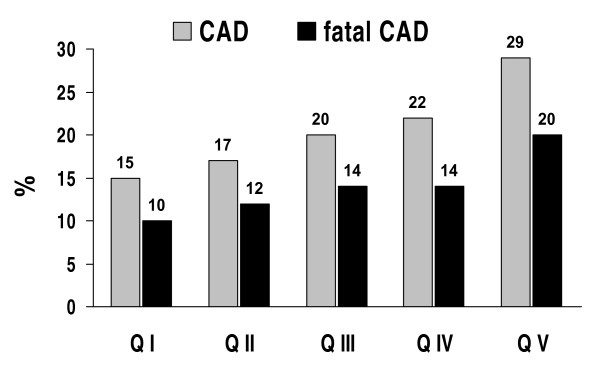
***UKPDS Risk Engine *10-year absolute predicted risk of developing non-fatal or fatal coronary artery disease (CAD; *grey bars*) or fatal CAD (*solid bars*) in 331 T2DM males according to quintiles of *log*(TG)/HDL-C ranking distribution**. Within each quintile, data were obtained from subsets of patients in primary cardiovascular prevention: QI: *n *= 83; QII: *n *= 59; QIII: *n *= 65; QIV: *n *= 65; and QV: *n *= 59. HDL-C: high-density lipoprotein cholesterol; TG: triglycerides. Significance of differences between quintiles: *P *< 0.0001 (CAD) and *P *= 0.0004 (fatal CAD).

In the whole cohort, 331 patients were in primary CV prevention, and therefore eligible for UKPDS risk estimation. Their mean 10-year absolute CV risk prediction was: 22 (16)% (CAD); 15 (14)% (fatal CAD); 14 (19)% (stroke) and 2 (3)% (fatal stroke). Figure 2 depicts UKPDS risk for CAD after adjustment for inter-quintile differences in mean age. There were significant trends for a markedly heightened risk across AD quintiles, from 15% (CAD) and 10% (fatal CAD) in Q I up to 29% (CAD) and 20% (fatal CAD) in Q V (p < 0.0001 and 0.0004). No such trends across quintiles were observed for risk of stroke or lethal stroke (*not illustrated*).

## Discussion

The main findings of the present study are fourfold. Firstly, we observed a 35% prevalence of AD in T2DM males. Secondly, the AD ratio *log*(TG)/HDL-C proved an easy means to estimate IR, and was strongly associated with IR and its cardiometabolic phenotype, including fat distribution, liver steatosis and sedentarity markers. Thirdly, the ratio was linked to a marked gradient for residual risk of future CAD. Fourthly, the ratio related to insulin secretion and β-cell function loss rate in T2DM, and as such a higher ratio was associated with non-attainment of target HbA_1c_. This AD ratio is therefore exquisitely suited to assess both non-LDL residual vascular risk and glucose homeostasis determinants in diabetic patients.

In the common form of T2DM, i.e. associated with MetS and IR, the hallmark of AD is decreased HDL-C level together with raised TG, while LDL-C is often only marginally elevated. Further to these obvious non-LDL abnormalities, there usually coexists an array of quantitative and qualitative lipoproteins abnormalities, not evidenced from standard lipid assessment. These include (*i*) a decrease in LDL size, often obscuring the absolute increase in LDL particles number; (*ii*) an increase in fasting and/or postprandial TG-rich apoB_48 _and/or apoB_100_-carrying lipoproteins and their remnants; (*iii*) a relative shift toward hepatic overproduction of apoC_III_-carrying VLDL and (*iv*) detrimental changes in HDL quality and natural history (eg. lesser atheroprotective subclasses, TG enrichment, reduced half-life and decreased nascent HDL production) [2,5,12,13,36-39].

In the present study, current TG levels, non-HDL-C levels, non-HDL-C *minus *LDL-C, and the ratio of LDL-C to apoB_100 _confirm the high prevalence of AD-related abnormalities in this mostly North-Caucasian population of T2DM males. AD was also associated with high number of apoB_100_-carrying particles and with markers of lipoprotein TG-enrichment [2,12,36,37]. Using consensual MetS cutoffs for defining decreased HDL-C and elevated TG [11,16], we observed a high prevalence of AD in T2DM males, at more than one-third. AD is however more likely to behave as a continuous CV risk variable. This underlies the rationale to use the combined occurrence of low HDL-C and high TG as dichotomic variable in order to assess AD *prevalence *in a population, while the *log*(TG)/HDL-C appears more appropriate to rank AD severity as a *continuous variable *in a given individual [12]. This is confirmed from comparison with values from control, non-diabetic subjects, showing that in T2DM, the AD ratio was well above normal values from the third AD quintile onwards, hinting to the fact that >60% within this population presents with some form of AD. This was not unexpected, TG and HDL-C being used to define the MetS phenotype, the latter being highly-prevalent (76%) of this T2DM population.

Whereas the *Atherogenic Index of Plasma *(*log*(TG/HDL-C) uses *log *transformation of the whole ratio, the present study only applied *log *transformation to TG numerator, as HDL-C range *per se *did not justify transformation. Skewness from untransformed TG/HDL-C distribution frequency was expected, with the TG term accounting for most of it. Whereas skewness of HDL-C frequency distribution was statistically significant, it was technically unimportant, and not surprisingly, HDL-C is rarely subject to prior *log *transformation neither in routine clinical assessment nor in clinical trials [12,17-20]. Dealing with a male-only population offered the advantage of avoiding bimodality in HDL-C and TG distributions due to gender differences (T2DM females exhibiting higher mean HDL-C and lower TG values (by an average 6 and 12 mg/dl, respectively; (data not shown)).

In order to rule out any confounding effect of LLD, we used *baseline *TG and HDL-C values to compute the ratio, prior to any anti-dyslipidemic drug(s), such as statins, fibrates or niacin, all of which being increasingly used in combination therapy for T2DM. The difference between baseline and current TG levels in this study averaged 33 mg.dL^-1^, supporting the rationale for such an unyielding approach to obtain *log*(TG)/HDL-C. This also allowed to establishing precisely the true, underlying magnitude and frequency of AD in T2DM. Regarding lifestyle-related confounders on AD prevalence, it is noteworthy that both smoking and ethanol intake showed significant decreasing trends across quintiles, the inverse association with ethanol being expected, due to its HDL-C-raising effects.

For non-lipid biochemistry the AD ratio was associated, in a stepwise gradation, with a series of emerging non-lipid cardiometabolic markers, all comorbid to T2DM, such as fasting insulinemia, _hs_CRP, uric acid, cystatin C, leucocytes count and glomerular filtration rate. SHBG and total testosterone, but not free testosterone, significantly decreased in parallel across AD quintiles, an expected finding since stepwise insulin resistance is associated with decreased levels of the androgen transporter [24,25,40-42].

IR develops in a conditional genetic or acquired environment associated with overall and central body fat, sedentarity, and skeletal sarcopenia. This array of metabolic abnormalities predisposes to CV risk, and is easily captured clinically by (*i*) the presence of a MetS phenotype and (*ii*) by the incremental MetS score [11,12,16,43,44]. Numerous abnormalities defining AD are associated, causally or as markers, with IR and its compensatory portal hyperinsulinemia, and with liver steatosis, the upstream organ responsible of excess VLDL synthesis and export [12]. The present results show that *log*(TG)/HDL-C is an easy means to rank T2DM patients alongside IR, liver steatosis, leisure-time sedentarity (with screen-watching as surrogate), lack of leisure-time physical activity, and MetS score. This extends previous findings showing that this ratio was a surrogate for IR in healthy African Americans and white Caucasians [45]. In our study, *log*(TG)/HDL-C also associated with both prevalent CAD, prevalent stroke but neither prevalent eye or nerve microangiopathy, nor stroke risk, nor prevalent PAD, the two latter caused by a combination of micro- and macrovascular end-organ damage. On the other end, albuminuria prevalence or level, both markers of mixed micro/macrovascular end-organ damage in T2DM, were significantly associated with AD.

In the *ACCORD Lipid *trial, impacting upon AD using a simvastatin-fenofibrate combination therapy reduced macrovascular risk in a subset patients with AD defined by concurrent HDL-C <1^st ^tertile *plus *TG >3^rd ^tertile of baseline cohort lipids. This was in line with previous beneficial impact of fibrates on macrovascular outcomes in *post-hoc *subgroup analyses from landmark fibrate trials [12,13,44,46-48]. By contrast, in the *ACCORD Lipid Eye *substudy, microvascular risk of retinopathy progression was markedly reduced by combination simvastatin-fenofibrate therapy in a broader range of baseline non-LDL lipids [49]. Besides fibrates, another promising pharmaceutical means to improve the AD ratio is to raise HDL-C levels with niacin or *cholesteryl ester transfer protein *inhibitors [5]. Parallel to pharmaceutical reinforcement, therapeutic lifestyle intervention emphasizing on a diet low in carbohydrates and ethanol, on caloric restriction and expenditure through exercise should not be overlooked as a means to improve the AD ratio, which in this study was clearly associated with sedentarity and lack of leisure-time exercise.

The AD ratio was also linked with more severe impairment in endogenous insulin secretion, as well as worsening of hyperbolic product (B × S) loss, heralding earlier and more intensive requirement for stepping-up of glucose-lowering therapies, including insulin [14,15,30,34,50]. This unexpected finding is all the more relevant, as the ratio simultaneously associates with more severe IR and faster insulin secretory loss. Such a detrimental combination is poised to lead to poorer glycemic control. This association between *log*(TG)/HDL-C and β-cell impairment does not simply reflect decreased insulin output due to β-cell loss, as the latter would bring about opposing effects on the ratio terms, with reduced liver TG synthesis and VLDL export (*portal *hypoinsulinemia) and reduced lipolysis of TG-rich particles (*systemic *hypoinsulinemia). In addition, systemic TG removal from VLDL may also be altered as a result of decreased insulin sensitivity affecting lipoprotein lipase [12]. Regarding glycemic control, T2DM patients with elevated raised AD ratio qualify for more proactive approaches to control hyperglycemia with insulin-sensitizers and/or insulin replacement therapy.

This study has several limitations. Firstly, we analyzed only male patients. Secondly, the cross-sectional design does not allow establishing causality relationships. Third, incorporation of routine measurement of HDL-C as denominator does not allow distinguishing dysfunctional, less atheroprotective or even atherogenic HDL subclasses which may be more prevalent in patients with T2DM. Besides compositional changes in HDL induced by overproduction of TG-rich lipoproteins [12], loss of functionality may also result from oxidative changes in predisposed individuals, such as those with haptoglobin 2-2 genotype, not captured from simple measurement of HDL-C level [51]. Nevertheless, dysfunctional subclasses often coexist alongside decreased HDL-C, so that a low HDL-C level in the ratio may reflect to some extent underlying qualitative defects [52]. Finally, the population under study was mostly of European Caucasian ancestry. The conclusions need to be confirmed in other major racial/ethnic diabetic and nondiabetic subgroups. The validity of the TG/HDL-C ratio as a simple, clinically useful estimator of IR was reported by Li *et al. *across race/ethnic subgroups in a large, nationwide sample of nondiabetic subjects from three major US populations (non-Hispanic whites, blacks and Mexican Americans) [53].

Based on the present results, we consider *log*(TG)/HDL-C as a simple means to estimate AD, and the residual vascular risk it confers, in fully-treated T2DM male patients. In addition, AD closely associates with the two major determinants of glucose homeostasis, affecting the natural history of hyperglycemia and its metabolic control. In patients in primary CV prevention, this AD ratio also yielded a marked, stepwise increase, in 10-year CV risk for CAD and fatal CAD. Using the *log*(TG)/HDL-C therefore adds a wealth of pertinent information on residual macrovascular risk, beyond that provided by individual components of the available lipid profile. In T2DM, this AD ratio is a simple means to assess both non-LDL-related residual vascular risk and glucose homeostasis.

## Competing interests

The authors declare that they have no competing interests.

## Authors' contributions

All authors read and approved the final manuscript: MPH collected and managed the T2DM patients database; MPH, SAA and MFR contributed equally to the study design, data and statistical analyses and to drafting the manuscript.
